# Food Insecurity in Higher Education: A Contemporary Review of Impacts and Explorations of Solutions

**DOI:** 10.3390/ijerph20105884

**Published:** 2023-05-19

**Authors:** Brittany M. Loofbourrow, Rachel E. Scherr

**Affiliations:** 1 Department of Nutrition, University of California, Davis, CA 95616, USA; bloof@ucdavis.edu; 2 Family, Interiors, Nutrition and Apparel, San Francisco State University, San Francisco, CA 94132, USA; 3 Scherr Nutrition Science Consulting, San Francisco, CA 94115, USA

**Keywords:** college, food insecurity, supplemental nutrition assistance program (SNAP), COVID-19

## Abstract

Food insecurity is a global phenomenon which impacts a variety of social, economic, and life-stage groups. One such group affected by food insecurity is college students, who tend to experience food insecurity at a prevalence which exceeds the average of their local communities. The impacts of food insecurity in this population are multifaceted and have implications for their college experience and beyond. Food insecurity has been observed to have negative effects on college student academic performance, physical health, and mental health. This review explores the impacts of and solutions for food insecurity in this population globally, with particular emphasis on the United States, and specifically California.

## 1. Introduction

Food insecurity, the lack of access to nutritionally adequate food to support a healthy and active lifestyle, is a concern for a significant proportion of the United States (U.S.) population. In 2020, 89.5% of U.S. households were considered food secure; of remaining households, 6.6% experienced low food security (uncertain access to quality foods) and 3.9% of households experienced very low food security (possibly disrupted eating patterns) [1,2]. While these proportions maintained the previous year’s general outlook on food security in the US, this represents over 40 million individuals experiencing some level of food insecurity throughout the year [2]. It is also a growing concern in the college student population that has garnered much attention within the last fifteen years [3,4,5,6,7,8,9,10,11,12,13,14,15,16,17,18,19,20,21,22,23,24,25,26,27,28,29]. In 2020, college students numbered 19.4 million students, representing 41% of 18- to 24-year-olds, making this group a nontrivial subpopulation in the US [30,31]. Food insecurity is typically described in terms of broad demographic groups: age groups including children, adults, and seniors; racial/ethnic groups including white non-Hispanic, Black non-Hispanic, Hispanic, and Other non-Hispanic; household composition, such as married couples and single-parent households; and residential characteristics, including metropolitan area and geographic region [32]. While these broad categorizations are extremely valuable, they fail to take into account subgroups of individuals whose experience may not be widely shared and thus become invisible problems in the eyes of the general public. College students have a unique set of circumstances that may alter their food security, while also sharing characteristics which may contribute to their food security status, including factors like income level, race, location, whether they are first-generation college students, and whether they transfer to a 4-year college [33,34,35,36].

The following presents a contemporary review of the literature of food security at the college level. Particular emphasis has been placed on institutions of higher education in the US and California specifically, which hosts the largest number of colleges in the US. Selection criteria for inclusion in this review includes articles published within the past 15 years, which prioritize the college experience of food security as it relates to student finances, academic performance, physical and mental health, food choice, the COVID-19 pandemic, and efforts to improve student food security.

## 2. The College Food Security Landscape in the US and California

College students have often been considered to be of a “privileged” or “elite” group, however many across the country, including those enrolled in private universities, struggle with food insecurity [37,38,39,40,41]. Students are a group which is highly impacted by food insecurity; prevalence estimates on campuses range between 19% to 56%, with many campuses reporting food insecurity prevalence around four times the national average [42,43,44,45,46]. Regardless of perceptions of college students, food insecurity is a pervasive issue which touches large proportions of students from all backgrounds [47].

Food insecurity in California persists regardless of the state’s large agricultural output, and this experience trickles down to affect students on the 116 campuses of the California Community Colleges (CCC), 23 campuses of the California State University (CSU), and 10 campuses of the University of California (UC) [48]. California students have been observed to experience food insecurity at the same disproportionately high prevalence as other colleges nationwide, with a study of the UC indicating that about 44% of its student population experience food insecurity [49]. Similar to the national distribution of food insecurity, prevalence changes depending on campus location [50]. The average food insecurity of all types of institutions reflect these differences, with average food insecurity prevalence at CCCs being of 52% and CSUs being estimated at 21% [50,51]. The prevalence of food insecurity at these institutions tracks generally with students who are from low-income backgrounds, with just over half of CCC students, and 40% of CSU and UC students being from low-income backgrounds [50,52]. Other students who frequently experience food insecurity are first-generation students (those whose parents did not attend a 4-year college) [53]. Consistent observations of food insecurity among these demographic groups contribute to observations of poor college readiness [48,49,50]. Students who are low-income and/or first-generation reportedly do not have the same level of readiness to attend college and function with autonomy in a new environment as peers who do not come from low-income backgrounds and whose caregivers attended college [54]. This lack of preparedness and ability to manage one’s basic needs, such as food procurement and preparation, can affect not only their academics, but it may also have implications for students after they leave the college setting and must support themselves outside of school [54].

## 3. Global Food Insecurity among College Students

In other countries where student food insecurity is studied, the prevalence of food insecurity on college campuses appears to mirror that of the US. Food insecurity in college remains high, with research from Australian universities indicating a food insecurity prevalence of 38–48% among students [55,56]. Similarly, a study of public and private college students in Lebanon indicated that food insecurity affected 39% of surveyed students [57]. As has been observed in US university students, food insecurity has been associated with poorer academic outcomes, with Malaysian students experiencing food insecurity having a lower GPA than food secure colleagues [58]. Regardless of university location, students who attend universities outside of their home country tend to experience higher prevalence of food insecurity, due to factors like lack of family support, culturally appropriate foods, and high costs of foods [59]. A group of international students experiencing food insecurity at a Canadian university indicated that knowledge of local and culturally appropriate foods impacted food security and thus, their overall academics [60]. International and domestic students experiencing FI both used similar coping mechanisms to deal with food insecurity including delayed bill payment, applying for loans, and working more. However, international students were less likely to ask friends or relatives for assistance [61]. A similar prevalence of food insecurity has been observed in students in Saskatchewan, with 39.5% of students experiencing food insecurity. Not dissimilar to what is seen in the US, students experiencing food insecurity were overrepresented by students relying on student loans as their primary source of income [62]. At another university in Canada, it was observed that over 35% of students experienced food insecurity, with indigenous students in particular being more likely to experience severe food insecurity [63]. While international students are not a homogenous group, trends in food security prevalence indicate that lack of culturally appropriate foods and a robust social support system may contribute to this experience.

## 4. Student Finances as a Factor in Food Insecurity

In the US, financial status and food insecurity have been observed to be very closely linked; students who are from low-income backgrounds are more likely to experience food insecurity [64,65]. Further, research in college students indicates that financial literacy (the knowledge and skills of personal financial management) is variable but limited [66,67,68,69]. Whether financial literacy is high in an individual to some degree is irrelevant, as the costs associated with college attendance—which include housing, health, transportation and food costs—are exceedingly high [70,71,72]. A student may have adequate knowledge and skills to manage their finances, but in the face of a myriad of financial constraints, this knowledge may not be enough to keep students from excessive financial burden. With the federal Pell Grant failing to cover most of the cost of college, public school budget cuts leading to more students paying higher tuition and fees, and debt being taken on to cover costs while attending school full-time, the ability to balance a personal budget is not enough to maintain financial stability [71]. In addition, a greater proportion of students are attending college from low-income backgrounds, widening the gap between financial stability and college attainment [52].

Although financial literacy is especially poignant in the context of food security and food literacy, it will not change the means that a student has available to them [73]. Food literacy (the knowledge, skills, and behaviors needed to manage one’s dietary intake) and food security are both partly dependent on financial literacy [74]. The ability to procure and prepare foods is predicated on the ability to prioritize money for foods, and limited financial literacy combined with low means may inhibit food security and stymie food literacy before it is able to develop in this group [74].

## 5. Food Insecurity and Poorer Academic Performance

College food insecurity is frequently observed to have a negative association with academic performance. In the college student population, academic performance is a critical outcome area, an idea which is supported by the plethora of research articles describing how food insecurity affects GPA [13,25,27,34,42,75,76,77]. Camelo and Elliot showed that food insecurity is negatively associated with GPA, both alone and when considering demographic covariates (including race/ethnicity, age, Pell grant eligibility, and academic year) [78]. Further, their study demonstrated that food insecurity was a partial mediator of race/ethnicity’s association with GPA, which the authors describe as one way that achievement gaps observed among groups may persist [78]. Van Woerden et al. found that GPA differed between food secure and food insecure students by a startling 0.25 grade points [36]. Brescia and Cuite found that in addition to students experiencing low and very low food security, those experiencing marginal food security had a decrease in GPA compared to those with high food security while accounting for demographic factors like age, citizenship, race, enrollment and first generation status, and financial factors like grant receipt and family support [79]. While the most common metric is GPA, others have cited retention and neglect of academic responsibilities as other correlates with food insecurity [80,81]. Phillips et al. report that students experiencing food insecurity are about 3.5 times more likely than their food secure peers to consider dropping out of school, and about 3 times more likely to neglect academics in favor of earning a wage to support themselves, when comparing similar demographic and financial characteristics [81]. Similar results were observed by Wolfson et al., with results compounded by students who were the first in their family to attend college [80]. These results were echoed in a survey of students experiencing food insecurity in New York, who reported a decreased ability to do schoolwork in the early months of the COVID-19 pandemic after adjusting for race/ethnicity, age, degree program, and household size [82]. Reasons behind this association are likely complex and varied; studies have pointed to the ways that food insecurity is associated with poorer physical health, poorer sleep, and poorer mental health [76,83]. Mental health in particular has been implicated for its role in academic performance. Studies have shown that food insecurity is directly correlated with poorer mental health, which is linked to subsequent decreases in GPA [76,77]. Students have described this aspect of food insecurity and poor performance as taking “a lot of mental power,” causing academic strain due to inability to concentrate and the ways that the sensation of physical hunger can impact academics by increasing fatigue and lowering stamina [75]. Stebledon et al. conducted similar qualitative evaluations of food insecurity on campuses and found sentiments from students which echoed these, with one student indicating feelings of poorer mental and physical health during times of worse food security [84].

The consistent observation of the association between food insecurity and academic performance indicates a clear and actionable area for colleges and universities to prioritize providing support for their populations [84]. Many institutions have recognized the utility of promoting food security on campus and have allocated resources accordingly [85]. As pointed out by Stebledon et al., food insecurity is a factor in academic performance which can be modified; by supporting students food security, institutions of higher education may improve their own rankings [84].

## 6. The Physical and Mental Health Toll of Food Insecurity

Food insecurity has been associated with health concerns like overweight and obesity, and long-term chronic diseases like type II diabetes and cardiovascular disease [86]. In the college student population, studies have indicated that students experiencing food insecurity confront similar health issues [87]. The prevalence of overweight/obesity in this population was near 40% according to the 2020 American College Health Association’s National College Health Assessment [88]. Work by Huelskamp et al. indicated that food insecurity was linked to possibly obesogenic food strategies like eating more than normal when food was plentiful, attending events that offer free food, and eating more processed and cheap food in order to eat more [89]. Students participating in a qualitative study about the experience of food insecurity corroborated these findings by indicating that the experience was associated with both weight loss and weight gain, attributed to lack of high-quality food in their on-campus dining options [90]. Additionally, a recent study by Knol et al. found that students experiencing food insecurity were more than twice as likely as food secure students to report fair/poor general health compared to excellent/good health when adjusting for demographic and lifestyle characteristics like gender, race/ethnicity, academic class level, and financial situation [91]. Additionally, Martinez et al. indicate that food insecurity is associated with poor health, increased BMI, fewer days of enough sleep, less exercise, and fewer daily servings of fruits and vegetables when considering race/ethnicity, sex, history for food insecurity, financial aid, being an undergraduate student, and campus affiliation [83].

As previously mentioned, another area of health that has associations with food insecurity is poorer mental health [42,82,92,93]. Raskind et al. showed that food insecurity was associated with higher anxiety and depression and lower hope when controlling for socioeconomic and demographic factors, including gender, age, race/ethnicity, type of school attended, parental education level, living situation, employment, and Supplemental Nutrition Assistance Program (SNAP) participation [77]. Diamond et al. described that both short- and long-term food insecurity are associated with symptoms of depression, stress, isolation, and poorer resilience when accounting for demographic variables, including identifying as a member of the LGBTQ community [94]. Zickgraf et al. found that alongside anxiety and depression, food insecurity was linked to eating disorders, even when considering the effects of anxiety and depression, and including demographic covariates and socioeconomic background [95]. Both food insecurity and low fruit and vegetable intake were associated with depressive symptoms in a study by Wattick et al. [96]. In a study of Lebanese college students, accounting for demographic and socioeconomic factors, food insecurity was linked with higher depression and anxiety scores compared with food secure peers [57]. In a recent study, Oh et al. found links between food insecurity and substance use, including higher odds of binge drinking, cigarette smoking, and other illicit or prescription drug use after adjusting for demographic and employment factors [97]. Food insecurity has also been observed to be associated with a lack of social connection, which has been implicated in mental well-being [98]. Oh et al. found that in addition to increased loneliness, food insecurity was associated with greater odds of self-injurious behaviors, including a more than double likelihood of attempting suicide [99]. Although the literature in this area continues to grow, relatively few studies exist to show the impact of food insecurity on health in this population; more research is merited to illustrate the relationships between food insecurity and health outcomes in college students. One area of research which may build out this picture of food insecurity and health outcomes is the study of the drivers of student food choice.

## 7. Motivators of Student Food Choice

Food choice is a complicated issue, comprising many motivators that vary in importance depending on individual circumstances [100,101,102]. In adults, factors considered in food choice can include hunger, personal identity, social connectedness, nutrition, knowledge and skills, habits, and many others [103,104,105,106]. Time commitments for class responsibilities may limit time to prepare and eat foods [7]. Food choice constraints including convenience and cost have also been identified as motivating factors [107,108]. In the more general college population, food choice motivators are less well-understood. The lack of understanding may be compounded by the nuance of a student’s background and personal preference and a new eating environment. Some students may have meal plans that allow them to eat foods on campus, but the availability and adequacy of meal plans does not guarantee that a student will have access to foods that meet their needs, nor does it guarantee their food security [109].

The dearth of literature in this area has led researchers like Vilaro et al. to work toward building a scale to assess college food decision-making [110]. This study found that food choice was influenced by a myriad of factors, including social media and advertisements, health, quality, effect on body appearance, taste, cost, convenience, familiarity, and how filling the food was [103,110]. Although this study contributes meaningful results to build out the picture of student eating patterns, it does not address how food insecurity may relate to food choice. Other work has identified differences in fruit and vegetable intake between students who are experiencing food insecurity and those who are not [111]. A study by Tallant indicated that first-year students’ food choices change after taking a nutrition seminar, with a majority of students reporting healthier food choices and more nutrition label reading following a 16-week nutrition course [112]. To add to the picture of differences in food behaviors, a study by Knol et al. showed that food preparation skills and feelings of cooking self-efficacy were different between students experiencing very low food security and those considered food secure [113]. Together, these studies create an unclear but compelling picture of the ways food insecurity and food choice interact. More studies are merited to establish drivers of food choice in college students, in order to both learn more about the ways that diet quality differs and to leverage those results in building programs which support student diet quality.

## 8. Ways of Promoting Student Food Security 

### 8.1. Means-Tested Financial Aid for Students in Need

One solution to promoting food security is by providing students from low-income backgrounds with financial aid to offset the costs of foods. These means-tested financial aid sources may be distributed from the federal government or state government, and include grants like the Pell Grant and CalGrant, respectively [114,115]. In order to receive these grant funds, students must be eligible by demonstrating financial need (a student’s cost of attendance compared to their expected family contribution), be a citizen or eligible noncitizen, be enrolled at least half-time, and other criteria [115].

Despite monies being distributed to students exhibiting financial need, receiving grant funds does not appear to be protective against experiencing food insecurity [116,117]. Research indicates that students who are Pell Grant eligible or Pell Grant recipients are significantly more likely to be food insecure [37]. This correlation likely points to a larger problem in student finances and the cost of college; grant funds are not enough to lift students out of financial instability and ensure their basic needs are met [72]. Students who receive need-based grants are likely still financially unstable, and those who receive these grants remain food insecure [116]. Recent research at the UC indicates that students have identified high college costs as one reason for decreased food security. Tuition and fees levied by college institutions consume student financial aid, such that students are unable to use financial aid for basic needs like food in lieu of paying for schooling costs [118]. Although means-tested financial aid is a great resource for supporting low-income and first-generation college students, these funds are not enough to support the full costs associated with college attendance, leaving students with limited resources likely to sacrifice their housing and food security as their financial aid monies are claimed by their college institution [118].

### 8.2. Campus Food Pantries as an Emergency Response to Food Insecurity

Food pantries are expanding across the US [119,120]. The College and University Food Bank Alliance (CUFBA, now Swipe Out Hunger) is a professional organization of on-campus food pantries and support, which has consistently reported growth in its membership as student food security concerns are highlighted [121]. According to Swipe Out Hunger, membership in the organization grew from 262 pantries in 2018 to over 700 as of early 2021 [121,122]. Chief among these efforts are food pantries which are supplied through university efforts, partnerships with local food banks, purchasing foods from grocery stores, and others [122]. These exist with the aim of supporting food security in student populations, however their distribution across institutions is uneven [123]. Implementing a food pantry on a college campus can be exceptionally challenging, with cited barriers including securing staffing and volunteers, and a lack of clarity in establishing a new pantry [123]. To support campuses interested in implementing a pantry, Swipe Out Hunger has toolkits available which describe starting and running a pantry, however these resources do not solve the problem of a lack of funds, staffing, or perceived legitimacy of pantry efforts [121,123]. However, the growth of Swipe Out Hunger membership indicates that college food insecurity is becoming more visible on campuses, and that there is a growing interest in supporting students’ basic needs [121,124].

Many campuses in California have developed programs to help promote student food security. At the UC, pantries have been established at all ten campuses, and funds have been dedicated from the California state budget to support these establishments [85,125,126]. The UC estimated that 52,000 students were served across its campuses in fiscal year 2017–2018, however this estimate may be only 30% of students experiencing food insecurity [126]. At the CSU, all 23 campuses offer a food pantry [127]. Although termed “vital” by students, a 2019 report of CSU basic needs programs estimated that just 16.7% of food insecure students participate in these resources [128]. At the CCC, nearly all of the 116 campuses serving nearly 2 million students across the state have an on-campus pantry [125].

Unfortunately, a campus having a food pantry does not guarantee that all students experiencing food insecurity will use the resource. Research has indicated that participation in pantries by students varies, and that barriers include factors like perceived stigma and conflicting self-identity, as well as logistical barriers including lack of knowledge about the resource and time conflicts [90,129,130]. To help address these areas, the Hope Center recommends that lecturers add a statement about food security and available resources to their course syllabus [131]. In addition to spreading knowledge about resources, stigma surrounding food insecurity and utilizing food pantries may be reduced by normalizing the open discussion of these topics [131]. A study by Esaryk et al. supports this idea by showing that open discussion of resources by on-campus food pantry staff resulted in more visits to the pantry [132].

Although campus food pantries may help to support student’s food security, it is important to note that the goal of these organizations is not to guarantee food security or be relied upon for the long term [123]. To that end, institutions seeking to improve campus food security can provide students with connections to other programs that may supplement campus resources, including federal programs like SNAP.

### 8.3. National Programs Can Promote Student Food Security

While localized efforts are helpful, federal entitlement programs may also be beneficial. The Supplemental Nutrition Assistance Program (SNAP) is the largest social welfare program in the U.S., providing an average of $166 monthly per each of its near 40 million participants [133,134]. Research regarding SNAP typically focuses on adults of various subgroups throughout the US, with delineations in data occurring at the demographic, geographic, and health outcome levels. College students remain a highly underrepresented group in these studies. 

In California, research has indicated that many students do not participate in CalFresh (the name of SNAP in California), although eligibility requirements for college students may allow for a significant proportion of students to participate [135]. According to the California Department of Social Services, over 416,000 college students across the state are likely to be eligible to participate in the program [136]. In spite of this wide eligibility, only 127,360 students receive CalFresh benefits annually [136]. Due to an overall lack of representation of college students participating, it is unclear what the main driver of nonparticipation is. In some populations, stigma associated with welfare programs like SNAP have been cited as barriers to participation [137]. This stigma about social safety nets may include assumptions that participants are lazy, that they do not or cannot hold a job, associations of poverty with decreased quality of life, not wanting a “hand-out,” concerns about outside perceptions and embarrassment, and shame [137,138].

In the college student population, a key barrier cited by researchers are unclear eligibility requirements [72,139,140]. In order to qualify for CalFresh benefits, college students must meet one of several criteria, including meeting income requirements, working an average of 20 h per week, participating in programs that increase their employability, or receiving federal Work Study [135]. These criteria are cited as being confusing for students, if they are known at all [72]. To address this barrier, colleges in California have partnered with the state in recent years to streamline student eligibility and make eligibility clearer [126]. If students at the UC participate in work study or receive the federal Pell Grant, they receive an automated message indicating their possible eligibility and a link to a verification letter to present to CalFresh eligibility workers at the county level [141]. California colleges continue to partner with the state to find solutions to make CalFresh more accessible [142]. By improving access to this program, participation rates may increase and boost food security in this population.

## 9. Food Security Concerns in Response to External Shocks: COVID-19 

The circumstances surrounding COVID-19 were unprecedented for college students [143]. Early in the pandemic, campuses nationwide closed to students, forcing many to return home or maintain housing local to the university [144,145]. Alongside this change in housing, some students also reported a change in work, as part-time work evaporated when businesses shut down in response to local lockdowns [144,146]. Literature regarding details of how this impacted students is still emerging, but early results indicate that the pandemic precipitated significant increases in food insecurity [146,147,148]. Barber et al. conducted a study at UCLA examining the relationships between remote learning, food insecurity, first-generation status, and under-represented minority status, and found that the transition to remote learning due to the pandemic had significant negative impacts on food security in these groups [53]. Owens et al. conducted a study at a Texas university which indicated that changes in living arrangements and job status due to the pandemic were strong predictors of food insecurity, highlighting the tenuous position many students are in with limited resources [144]. Eating patterns were also observed to change, with dietary patterns in surveyed groups of students at universities in Texas, with results indicating fewer weekly servings of fruits and vegetables during the pandemic [149,150]. Ahmed et al. described worsening food security in New York colleges, however there were increases in students’ knowledge about food resources and willingness to use the resources during the pandemic compared to pre-pandemic measurements [151].

An analysis of psychosocial health and food insecurity during the COVID-19 pandemic by DeBate et al. found that students experiencing food insecurity also experienced poorer mental health and lower levels of resilience and flourishing [152]. The authors pose that universities have a responsibility to their students to address food insecurity such that in the event of public health emergencies like the recent pandemic, these students do not struggle disproportionately more than their peers [152]. Similar calls to build emergency preparedness at the campus level were echoed in a study by Silva et al., who found that student diet quality decreased during the pandemic lockdown, and particularly so for students experiencing food insecurity [153]. Bergdahl et al. found that for students experiencing food insecurity, government supports in the form of stimulus payments to students from low-income backgrounds were most helpful for alleviating food insecurity [154]. Federal stimulus payments were also observed to be of particular benefit to students when combined with SNAP benefits at a large California university [155]. Unfortunately, resource availability was not stable for all college students nationwide; such differences in resource availability for vulnerable students may exacerbate inequities and compound negative experiences during times of acute stress or emergency [156].

## 10. Conclusions

Research in the area of food insecurity in college has expanded in vast and meaningful ways since its early explorations [5]. Dozens of studies have evaluated the prevalence of food insecurity across US college campuses and found that college students are not exempt from the negative health associations seen in other populations [47]. Students experiencing food insecurity have been observed to report poorer physical and mental health, and consume fewer foods associated with healthful eating patterns [83]. Limited research has explored associations of food insecurity with poorer quality dietary patterns by exploring food choice motivation, and of particular concern how the frequently accompanying financial insecurity may influence food choice [108].

It is imperative to find ways to fill the gaps in food security and provide students with healthful foods which support their physical health, mental health, and academic performance. Resources are available to different degrees at the campus level with expanding access to food pantries nationwide. Unfortunately, participation in these resources has not been observed to be high, even among students experiencing food insecurity. Stigma associated with food pantries may be a reason for low participation, but increasingly limited knowledge and open dialogue about food insecurity and resources appear to be a driver for low participation [130,131,132]. Important resources which may be of great benefit to this population are CalFresh/SNAP benefits, as the program allows participants to use funds to select foods that help meet their own needs and wants. 

In the context of COVID-19, food resources were of particular importance. Due to campus closures, many students’ living situations changed, and many were unable to utilize on-campus resources as they may have otherwise [156]. In this unprecedented public health emergency, the utility of CalFresh is highlighted; during the COVID-19 pandemic, program benefits were expanded to support food security in vulnerable individuals in the face of widespread lockdown and job loss [157].

Under normal circumstances, research indicates that SNAP is effective in supporting food security in the general population, and although this research has not been conducted in college students, it logically follows that this population would experience similar benefits [158]. When considering the ebb and flow of college academic calendars, the consistent benefits available through a federal food support program are critical not only in emergencies, but also during normal times when on-campus resources are not available, such as holiday and summer breaks [148]. Considering campuses which may not have robust food support programs like pantries available to their students, promotion of federal benefits may offer another avenue through which campuses can work to promote food security for their student body [72,132].

The effects of food insecurity are far-reaching, and although much has been and continues to be done to characterize this experience in college students, relatively little has been done to examine solutions to food insecurity and how these solutions may influence student physical health, mental health, and academic performance [72]. Given the concerns of food insecurity, greater research emphasis should be put on assessing the availability of food resource programs for students and more broadly examining ways to support student food access. Moreover, promoting campus-wide food security should be a priority for college administrators, in order to meet the needs of their students, maintain their reputations, and meet missions of excellence [84].

## Data Availability

Not applicable.
